# Carbon footprints by stage of chronic kidney disease: The case of Japan

**DOI:** 10.1016/j.joclim.2023.100294

**Published:** 2023-12-14

**Authors:** Kei Nagai, Sho Hata, Norihiro Itsubo, Kunitoshi Iseki, Kunihiro Yamagata, Keisuke Nansai

**Affiliations:** a Department of Nephrology, Hitachi General Hospital, 2-1-1 Jonan-cho, Hitachi, Ibaraki 317-0077, Japan; b Department of Nephrology, Faculty of Medicine, University of Tsukuba, 1-1-1 Ten-nodai, Tsukuba, Ibaraki 305-8575, Japan; c Material Cycles Division, National Institute for Environmental Studies, 16-2 Onogawa, Tsukuba, Ibaraki 305-8506, Japan; d Faculty of Environmental and Information Studies, Tokyo City University, 3-3-1 Ushikubo-nishi, Tsuzuki-ku, Yokohama, Kanagawa 224-8551, Japan; e Clinical Research Support Center, Nakamura Clinic, 4-2-1 Iso, Urasoe, Okinawa 901-2132, Japan

**Keywords:** Chronic kidney disease, Carbon footprint, Dialysis therapy

## Abstract

**Introduction:**

The nexus between carbon footprints and chronic kidney disease (CKD) progression have not been clarified, so it has not been possible to examine the prevention of disease severity as a potential countermeasure for decarbonization.

**Material and methods:**

The study included 70,627 subjects aged 40–74 years and diagnosed with CKD stage by specific health checkups in 2014–2015. Greenhouse gas (GHG) emissions in Japan were formulated with the 2015 environmental input–output model. The carbon footprints by CKD stage were calculated with annual treatment cost according to renal function indicators, namely estimated glomerular filtration rate (eGFR) and proteinuria.

**Results:**

The annual carbon footprint per patient with induction of dialysis due to CKD was estimated to be 3.9 tCO_2_e, in contrast to 0.31 tCO_2_e in subjects without dialysis. Highlighting the relationship between the carbon footprint and the slope of eGFR as the CKD stage advances, the carbon footprint of care for patients with a stable eGFR in CKD stage G2 or better was 300 kgCO_2_e in males and 280 kgCO_2_e in females. Yet, in CKD stages G3a and G3b or worse, the carbon footprint for a rapid decrease in eGFR (30 % or greater per year) was 620 kgCO_2_e and 1440 kgCO_2_e in males and 430 kgCO_2_e and 1270 kgCO_2_e in females, respectively.

**Conclusion:**

Effective interventional treatments to prevent disease severity in CKD contribute to both the health of the patient and the mitigation of GHG emissions.

## 1 Introduction

The concept of planetary health [1], which involves the recognition that human health is inseparable from a healthy global environment, is gaining increasing attention, and the direct and indirect environmental impacts of health-care provision have been quantified as environmental footprints [2]. Specifically, in 2015, health-care demand was responsible for 4.4 % of global greenhouse gas (GHG) emissions, 1.5 % of scarce water consumption, 2.8 % of particulate matter emissions, 3.4 % of nitrogen oxide emissions, and 3.6 % of sulfur dioxide emissions [3]. Healthcare accounted for 4.6 % of the total GHG emissions in Japan in 2011, with about half of these emissions coming from the demand for medical services due to hospitalization and outpatient care [4]. The decarbonization of health care has become increasingly important against the backdrop of the Paris Agreement (1.5 °C target) and the declaration of carbon neutrality by major countries [5]. In addition, at the G7 Health Ministers meeting in Germany on May 20, 2022, a commitment to support carbon mitigation through the health-care supply chain was also made.

Chronic kidney disease (CKD) is a risk factor for progression to end-stage kidney disease (ESKD), cardiovascular disease, and all-cause mortality, all of which generally require expensive treatment modalities and hospitalization [6]. The number of people currently receiving renal replacement therapy exceeds 2.5 million and is projected to double to 5.4 million by 2030, and the effect of CKD also expands well beyond the provision of renal replacement services [7]. Renal health care, especially hemodialysis therapy, is highly responsible for GHG emissions [8]. As an example, chronic hemodialysis in Australia was estimated to emit up to 10.2 tCO_2_ equivalent (eq) per person annually [9]. Similar carbon footprint studies on chronic dialysis treatments have been reported from the United Kingdom [10], China [11], and Japan [12]. This problem is significant as in Japan, one of every 380 people in the general population is on chronic dialysis because of ESKD [13], while one out of every eight to nine has CKD [14].

Clinical trials have traditionally been underpowered for the assessment of CKD outcomes that are infrequent or occur late because of the slow nature of the disease. Thus, the change in the estimated glomerular filtration rate (eGFR) is becoming a promising surrogate marker for clinical end points such as cardiovascular disease, ESKD, and all-cause mortality [15], [16], [17]. The early detection of CKD is available, and often inexpensive. Therefore, early interventions for CKD may be a promising strategy for improving renal and cardiovascular outcomes and delay or prevent progression to ESKD [18]. Still, the effects of maintaining renal function on climate change remain unknown. Specific health checkups are currently conducted throughout Japan and can be used as a tool to detect CKD high risk in the early phase. This helps with health promotion, and can facilitate large scale epidemiological studies from the early to advanced stages of CKD. We posited to use this data to document the association between changes in renal function and the amount of GHG emissions created by disease management.

## 2 Methods

### 2.1 Medical expenses of patients with CKD by annual changes in the eGFR

The medical costs for patients with CKD were determined in a previous study by the authors [19]; however, aspects of the study population are described to aid in understanding this study background. Japan's specific health check-up system is a public service provided to almost the entire general population aged 40–75 for the detection of lifestyle-related diseases and early intervention by public nurses. Specific health checkups are conducted separately from medical care and are not included in this treatment-related carbon footprint calculation for patients with CKD. The institutional review board for ethical issues of the University of Tsukuba approved this original study (No. 999; UMIN: 000019774).

The study population included 105,661 persons who had undergone annual specific health checkups in 2012 and 2013 [19]. Participants on chronic dialysis (*N* = 74) before the end of 2013, those not having consecutive medical checkups in 2013 (*N* = 34,283) and those with missing data (*N* = 677) were excluded [19]. The final group comprised 70,627 persons (54.2% female: age range, 40–74 years). Among these subjects, 128 males and 156 females had missing urinalysis data at baseline. The numbers of participants with each stage of CKD at the baseline year (2012) are presented in Table 1.

**Table 1:** Baseline CKD stage by eGFR in the subjects.

CKD stage at baseline	G2 or better	G3a	G3b or worse
eGFR, ml/min/1.73m^2^	≥60	45 to 59	<45
Male, number	26,030	5561	707
Female, number	32,948	4813	568

Abbreviations; CKD, chronic kidney disease; eGFR, estimated glomerular filtration rate.

The numbers of participants with negative proteinuria at baseline were 29,640 males and 36,644 females, those with proteinuria at baseline were 2530 males and 1529 females, and those with persistent proteinuria were 1329 males and 653 females. One should note that, persistent proteinuria was defined as positive proteinuria in both 2012 and 2013, so some cases of positive proteinuria and persistent urine protein may have overlapped. Annual change in eGFR was determined using data from 2012 to 2013, defined as [(eGFR in 2013 – eGFR in 2012) / eGFR in 2012 × (interval months / 12)] [19]. The numbers of subpopulations with a rapid decrease (decreases in eGFR of 30 % or greater per year) were 776 males and 1,412 females, those with a decrease in eGFR of 15 % to 30 % per year were 3,725 males and 5,388 females, those who were stable (decreases or increases in eGFR less than 15 % per year) were 25,321 males and 28,217 females, and those with an increase in eGFR of 15 % or greater per year were 2,476 males and 3,312 females.

### 2.2 Carbon footprint per unit medical expense

The carbon footprint per unit expenditure on hospitalization and non-hospitalization in Japan was calculated by converting the medical costs for patients with CKD into the direct and indirect GHG emissions associated with the costs. Because the carbon footprint of health care is larger for indirect emissions in the upstream supply chain than for direct emissions in hospitals [4], this study employed an IO-LCA (input-output based life-cycle assessment). An IO-LCA uses input-output tables as life-cycle inventory data, which describe annual transactions among production and consumption sectors in a whole economy [20], instead of collecting the inventory data one by one as in a process-based LCA. An IO-LCA has the advantage of being able to define clearly and capture comprehensively the upstream supply chain of the processes [21]. Furthermore, previous studies [4,22] have indicated that GHG emissions associated with fixed-capital formation, such as hospital construction and medical equipment, should not be neglected in the carbon footprint of healthcare services.

We formulated a capital-embodied input-output model as an IO-LCA method that combines the transactions needed for production activity and those for fixed-capital formation, as formulated in Eqs. (1) to (6). The target year was 2015, when the latest input–output table was available. In the 2015 Japanese input-output table, national economic activities are categorized into 390 economic sectors. Practically, many commodities correspond to the same sector. Therefore, it is important to note that commodities falling under the same sector have a higher carbon footprint in proportion to their prices.

First, we formulated the supply and demand balance of commodities in the Japanese economy, that is, outputs equal the sum of the intermediate and final demand, as Eq. (1) with the total output vector $x=(\begin{array}{c} x_{i}\\ x_{q} \end{array})$, the intermediate demand matrices $Z=(z_{ij})$, $B=(b_{ip})$, and $C=(c_{qj})$, and the final demand vectors and $y=(\begin{array}{c} y_{i}\\ 0 \end{array})$. These vectors and matrices were obtained from the 2015 Japanese input-output table. Here, $x_{i}$ and $x_{q}$ are the total outputs of commodity sectors *i* (*i* = 1…390) and fixed-capital formation sector *q* (*q* = 1…116), respectively, $z_{ij}$ (*j* = 1…390) denotes the intermediate input from commodity sector *i* to commodity sector *j*; $b_{ip}$ represents the intermediate input from commodity sector *i* to fixed-capital formation sector *p* (*p* = 1…116); $c_{qj}$ is the utilization amount of fixed-capital formation sector *q* at commodity sector *j*; and $y_{i}$ is final demand for commodity sector *i* . Note that because the supply chain is only domestic, imports are not included in the intermediate importance and final demand.

(1) $$x=\left(\begin{array}{cc} Z & B\\ C & 0 \end{array}\right)+y$$

Dividing the annual intermediate inputs by the total annual output, as shown in Eq. (2), gives the intermediate inputs per unit of output.

(2) $$A=\left(\begin{array}{cc} Z & B\\ C & 0 \end{array}\right)\times {\hat{x}}^{-1}$$

Where, A is the input coefficient matrix, hat (^) denotes a matrix with a vector as a diagonal element and the other elements are zero.

By substituting Eq. (2) into Eq. (1) to represent the intermediate demand matrix using the input coefficient matrix **A** and the output vector **x**, the amount of output can be determined according to the final demand as shown in Eq. (3).

(3) x=Ax+y

Transforming Eq. (3) into Eq. (4) and solving for vector x yields Eq. (5).

(4) x−Ax=(I−A)x=y

(5) $$x={\left(I-A\right)}^{-1}y=\mathrm{Ly}$$

Where, **L** $={\left(I-A\right)}^{-1}$ is the Leontief inverse where the elements of the matrix indicate how much a unit of intermediate demand for one sector directly or indirectly causes demand in other sectors.

Defining vector $d=(\begin{array}{cc} d_{j} & 0 \end{array})$ with GHG emissions per unit production of sector *j* as an element, Eq. (6) determines scholar *w* of the sum of direct GHG emissions directly associated with outputs x. Because final demand **y** generates **x** through *w* represents the GHG emissions induced by **y** or the carbon footprint. The elements of vector ***e*** = **dL** indicate the carbon footprint per unit final demand for each sector in the vector **y**.

(6) w=dx=dLy=ey

The 2015 Japanese input-output table defines the hospitalization and non-hospitalization sectors in the vector **y**. By obtaining the elements of vector **e** that correspond to these sectors, we determined the carbon footprint per unit expenditure for hospitalization and non-hospitalization in Japan. The data for vector ***d***, or sectoral-unit GHG emissions, were obtained from the authors’ previous study [23].

### 2.3 Carbon footprints per unit medical expense of hospitalization and non-hospitalization in Japan in 2015

The carbon footprint (ton CO_2_ equivalent: tCO_2_e) per unit expenditure (million Japanese yen: m-JPY; On average, 1 United States dollar = 121.6 JPY in 2015) was estimated to be 1.2 tCO_2_e/m-JPY for hospitalization and non-hospitalization in Japan in 2015. Of those values, fixed-capital formation caused 0.21 tCO_2_e/m-JPY and 0.16 tCO_2_e/m-JPY, representing 18 % and 14 % of the carbon footprints per unit cost, respectively. Onsite emissions of both hospitalization and non-hospitalization accounted for 0.11 tCO_2_e/m-JPY, which indicates that over 90 % of these carbon footprints occurs in the hospital supply chain. The main GHG-emitting sectors in the supply chain include power generation (0.47 and 0.48 tCO_2_e/m-JPY for hospitalization and non-hospitalization, same hereafter), freight and passenger transportation (0.14 and 0.16 tCO_2_e/m-JPY), pig iron (0.077 and 0.063 tCO_2_e/m-JPY), and cement (0.059 and 0.047 tCO_2_e/m-JPY).

## 3 Results

### 3.1 Health-care carbon footprints for patients with CKD

**Fig. 1:**
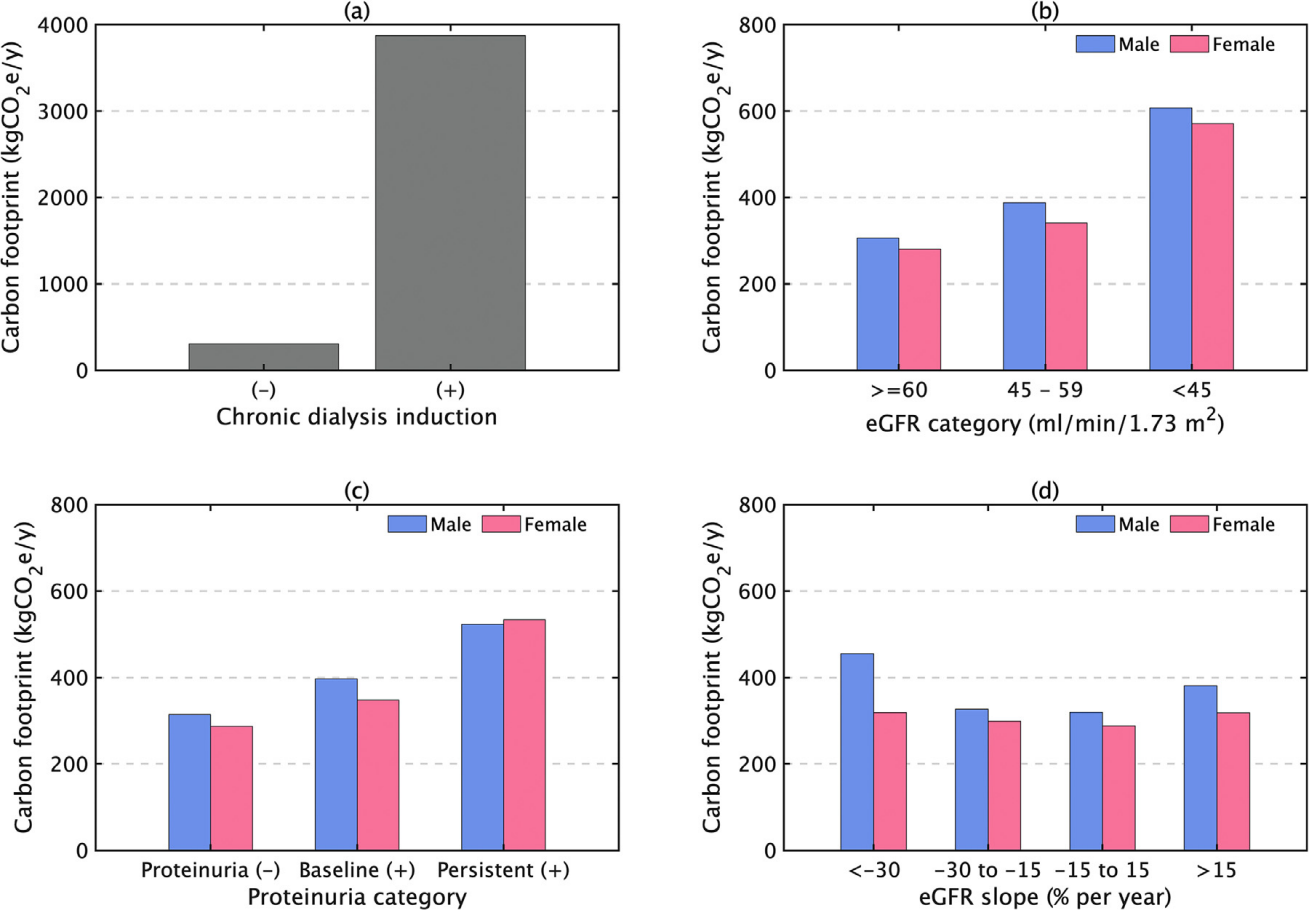
Annual capital-embodied carbon footprint per capita for chronic kidney disease (CKD). (a) Difference by with/without chronic dialysis induction, (b) Difference by renal function at baseline year based on the estimated glomerular filtration rate (eGFR: ml/min/1.73 m^2^) category (CKD stage 2 or better [≥ 60], CKD 3a [45–59], and CKD G3b or worse [< 45 ml/min/1.73 m^2^]), (c) Difference by proteinuria, and (d) Difference by slope of eGFR.

**Fig. 2:**
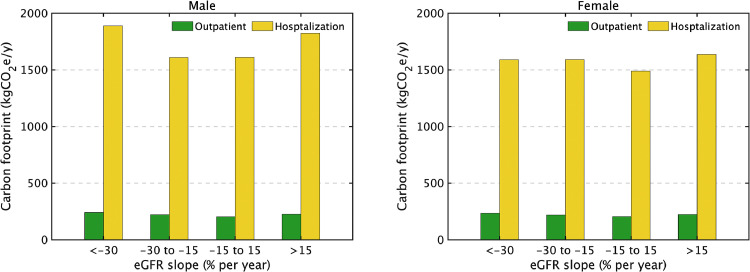
Annual capital-embodied carbon footprint per capita for chronic kidney disease (CKD) subdivided by the difference by slope of the estimated glomerular filtration rate (eGFR) and by the costs for outpatient visits and hospitalization.

The final cohort analyzed was 70,627 patients and 22 new dialysis inductions occurred in the 12 months after the evaluation period for renal function, representing 3.3 million yen in annual medical costs per person [19]. This amount was converted to a carbon footprint estimated at approximately 3.9 tCO_2_e emissions per person, in contrast to 0.31 tCO_2_e in subjects without induction of dialysis (Fig. 1a). Focusing on the presence or absence of CKD, i.e., eGFR or positive proteinuria, in the baseline year, the carbon footprints for health care among participants with CKD stage 2 or better (≥ 60 ml/min/1.73 m^2^) were 310 kgCO_2_e in males and 280 kgCO_2_e in females. These figures increased with the progression of CKD and doubled in CKD stage G3b or worse (< 45 ml/min/1.73 m^2^) (Fig. 1b). The carbon footprints for participants with negative proteinuria at baseline were 310 kgCO_2_e in males and 290 kgCO_2_e in females, and increased with positive proteinuria at baseline, and even more with persistent proteinuria (Fig. 1c). Furthermore, focusing on the rate of CKD progression, for participants with a rapid decrease in the eGFR, the carbon footprints were 1.4-fold higher in males and 1.1-fold higher in females, compared with a stable eGFR (Fig. 1d). A breakdown of healthcare-related GHG emissions showed that hospitalizations were around seven times higher than outpatient visits at any point on the slope of eGFR decline (Fig. 2).

### 3.2 Carbon footprints in the subpopulations based on the baseline and slope of eGFR

**Fig. 3:**
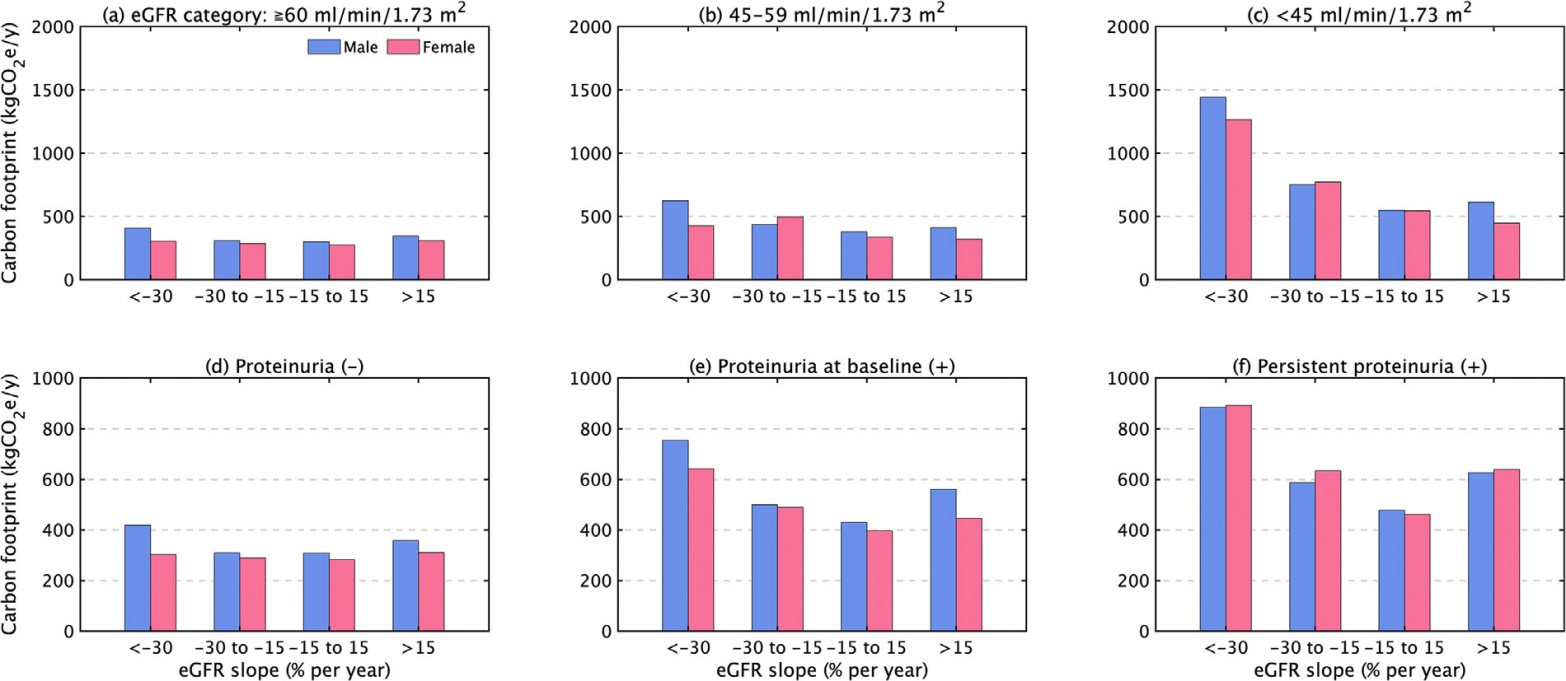
Differences in per-capita annual capital-embodied carbon footprint by renal function and proteinuria according to changes in the estimated glomerular filtration rate (eGFR: ml/min/1.73m^2^). (a) CKD stage 2 or better (≥ 60), (b) CKD 3a (45–59), (c) CKD G3b or worse (< 45) at baseline year, (d) Proteinuria (–), (e) Proteinuria at baseline (+), and (f) Persistent proteinuria (+) at baseline year.

The difference in carbon footprints among eGFR slopes is highlighted with advances in CKD stage. The carbon footprints per capita for the care of participants with a stable eGFR in CKD stage G2 or better were 300 kgCO_2_e in males and 280 kgCO_2_e in females (Fig. 3a). In CKD stages G3a and G3b or worse, the carbon footprints for a rapid decrease were 620 and 1440 kgCO_2_e in males and 430 and 1270 kgCO_2_e in females, respectively (Fig. 3b and c).

Compared with stable normal renal function, advanced CKD (G3 or worse) with rapid loss of renal function had four times the carbon footprint than did CKD stage G2 or better with a stable eGFR. The carbon footprints with a stable eGFR and negative proteinuria at baseline were 310 kgCO_2_e in males and 280 kgCO_2_e in females (Fig. 3d). With proteinuria at baseline and persistent proteinuria, the carbon footprints for a rapid decrease were 750 and 880 kgCO_2_e in males and 640 and 890 kgCO_2_e in females (Fig. 3e and f). This finding indicates that both proteinuria and baseline renal dysfunction have a strong impact on the increased carbon footprint due to the loss of renal function.

## 4 Discussion

### 4.1 Focus on carbon footprint through fixed-capital formation

This analysis of the carbon footprints for hospitalization and non-hospitalization in 2015 confirmed that the medical service supply chain creates greater GHG emissions than the direct emissions occurring at sites such as hospitals. This fact reaffirms the need for medical services to address how to reduce indirect emissions, with particular attention paid to emissions related to fixed-capital formation, those related to supply chains, pharmaceutical production plants and the power generation and materials industries. As previous studies [4,22] have pointed out, it is important to reiterate that hospital construction and repair represent major drivers of supply-chain emissions with regard to fixed-capital formation. The management of GHG emissions associated with fixed-capital formation is already a carbon disclosure item for industrial sectors, defined as category 2 (fixed capital) emissions in Scope 3, meaning the supply chain. The motivation for such carbon disclosures is to respond to the Task Force on Climate-related Financial Disclosures and the accountability of financial institutions for environmental, social, and governance investments. On the other hand, there are no financial incentives in Japan for reducing the carbon footprint of medical services. Financial institutions and government agencies, especially banks that provide financing and health administrations that regulate reimbursement, should play a role in requiring disclosure of Scope 3 emissions and decarbonization plans.

### 4.2 Less hospitalization and less disease severity as a decarbonization option

Although the carbon footprints per unit medical cost of inpatient and outpatient care were the same, the smaller costs for outpatient care reduces the footprint, which implies that preventing the exacerbation of disease and severity of illness necessitating hospitalization not only benefits patients, but also contributes to the decarbonization of medical services.

In the present case study targeting kidney diseases, we demonstrated that the carbon footprint required for treatment increases as the disease worsens. Specifically, the carbon footprint of treatment increases significantly as the kidney disease progresses a level requiring outpatient chronic dialysis treatment. Even in cases of moderate CKD stage G3b or proteinuria-positive cases without dialysis requirement, the carbon footprint is twice as high compared as that with non-CKD cases, which suggests that preventing severe renal disease is an effective measure for decarbonizing healthcare.

### 4.3 Association of diagnostic indicators with carbon footprints

As part of health check-ups, dipstick urine tests and eGFR measurement are established cost-effective screening methods in Japan, and CKD is diagnosed as a result of two consecutive years of positive proteinuria or reduced renal function (G3a or worse), because CKD is a chronic disease and urine protein tests are often transiently positive. To avert worsening of the disease, it is desirable to conduct yearly eGFR measurements and urine tests to screen for a rapid decline in renal function at an early stage, and provide diagnosis and intervention by a specialist physician. However, despite the high prevalence of CKD, which affects one in eight people, the number of individuals with rapid decline in renal function and classified as CKD stage G3b or lower is extremely limited, at less than 0.1 %. Moreover, only 0.3 % of those with positive urine protein experience a rapid decline. It is critical to detect these limited cases of CKD with rapid loss of renal function accurately. Recent collaborations on CKD between medical diagnostics and specialists/nonspecialists to prevent the deterioration of renal disease stage are expected to improve patient prognosis [24,25] and may contribute to decrease carbon footprints. Non-pharmacological and low-cost interventions such as nutritional and health guidance have been shown to slow the decline in eGFR and reduce the rate of cardiovascular events [26].

As a limitation, this study only employed an IO-LCA by extracting the volume of transactions required for healthcare from the input-output table, not-process based LCA. The difference in results from these two LCAs can be verified for dialysis therapy, and the 3.9tCO_2_e for maintenance hemodialysis patients in this study was very close to the 4.1tCO_2_e calculated previously by a process-based LCA on a field survey [12].

It is important for medical professionals to understand the relationship between diagnostic indicators of renal disease and carbon footprints as we pursue the decarbonization of renal disease treatment and healthcare in general. In addition, ensuring that patients understand the decarbonizing value of good health and less severe disease is fundamental for medical professionals in proposing preventive measures and treatments with low carbon emissions. Although this paper focuses on kidney disease, the value of linking carbon footprints to indicators for diagnosing patients is expected to rise for other diseases as well. Therefore, in the future, it will be vital to develop numerical information on carbon emissions required for treatment, as well as to educate health-care professionals on the significance of such information.

## Data sharing statement

Data are available upon request to the corresponding author.

## Funding

JSPS Grant No. 23K11528, AMED Grant No. 17ek0310005h0003/20ek03100010, and MHLW Grants-in-Aid for Scientific Research No. 23BA1002

## CRediT authorship contribution statement

**Kei Nagai:** Writing – original draft, Investigation, Funding acquisition, Conceptualization. **Sho Hata:** Writing – original draft, Investigation, Conceptualization. **Norihiro Itsubo:** Writing – review & editing. **Kunitoshi Iseki:** Writing – review & editing. **Kunihiro Yamagata:** Writing – review & editing, Funding acquisition. **Keisuke Nansai:** Writing – review & editing, Funding acquisition.

## Declaration of competing interest

The authors declare that they have no known competing financial interests or personal relationships that could have appeared to influence the work reported in this paper.
